# Investigation of Sleep Quality and Intolerance of Uncertainty in Liver Transplant Recipients: A Cross‐Sectional Study

**DOI:** 10.1111/nhs.70029

**Published:** 2025-01-20

**Authors:** Adile Savsar, Özgül Karayurt, Murat Kiliç, Gülay Aksu Kul

**Affiliations:** ^1^ Faculty of Health Science Department of Nursing Izmir University of Economics Izmir Turkey; ^2^ Liver Transplant Center Acibadem City Hospital Izmir Turkey; ^3^ Faculty of Medicine University of Economics Izmir Turkey

**Keywords:** liver transplantation, sleep quality, transplant recipients, uncertainty

## Abstract

Liver transplant recipients experience many uncertainties after transplantation. Also, sleep problems are common among them. This study aimed to examine intolerance of uncertainty and sleep quality in liver transplant recipients. A descriptive and cross‐sectional study was conducted with 117 liver transplant recipients followed in the outpatient clinic of a liver transplant center. Data were collected using a sociodemographic and clinical features form, the Intolerance of Uncertainty Scale‐12 and the Pittsburgh Sleep Quality Index between May and September in 2023. The data were analyzed using descriptive statistics, correlation analysis and regression analysis. The recipients had a high mean score of 41.04 ± 13.11 (min–max: 12–60) on the Intolerance of Uncertainty Scale‐12. Age was predictive of uncertainty. Indeed, young recipients had higher intolerance of uncertainty. The recipients had a low score of 7.33 ± 3.57 (min–max: 1–21) on the Pittsburgh Sleep Quality Index. The prevalence of sleep problems was 65%. Experiencing complications after transplantation was predictive of and worsened sleep quality. The sleep quality of the recipients had no relation with intolerance of uncertainty and inhibitory anxiety, but it had a relation with prospective anxiety.

**Summary**

- The quality of sleep was low and intolerance of uncertainty was high in the liver transplant recipients.
- The younger liver transplant recipients had higher intolerance of uncertainty.
- The liver transplant recipients experiencing complications and prospective anxiety had a poorer quality of sleep after transplantation.

**Abbreviations**
IUS‐12: Intolerance of Uncertainty Scale‐12
LT: liver transplantation
PSQI: Pittsburgh Sleep Quality Index

## 1 Introduction

Liver transplantation is a life‐saving treatment for patients experiencing complications from cirrhosis and those with stage T2 hepatocellular carcinoma (Terrault et al. 2023). It is reported that of all solid organ transplants performed in 2022, 37 436 were liver transplants obtained from cadavers and live donors and that liver transplants were the second most common transplants (WHO 2022). In Turkey, 1756 patients had liver transplants.

Although liver transplantation saves lives, liver transplant (LT) recipients can experience some physical and psychological difficulties after transplantation. Posttransplant treatments (especially immunosuppressive therapy) can negatively affect the quality of life (Yıldız 2021; Moayed et al. 2019; Taher et al. 2021). Due to various factors like organ rejection, side‐effects of medications, prospective anxiety, difficulties in relationships with people at work, family members and friends, and fear of nonadherence to medications, the recipients can experience anxiety, depression, uncertainty, and sleep disorders (Moayed et al. 2019; Taher et al. 2021; Yıldız 2021). In a meta‐analysis (2023), 28% of the LT recipients were shown to have sleep disorders in the posttransplant 1 year (Biyyala et al. 2023). Poor sleep quality can negatively affect the disease prognosis and healing (Zhu et al. 2020). Since sleep disorders are common in LT recipients, it is reported that further studies focusing on sleep are needed (Zhu et al. 2020; Lim et al. 2023; Biyyala et al. 2023; Cordoza et al. 2021). The studies conducted so far have revealed that gender (Bhat et al. 2015; Mendes et al. 2014), body mass index (Reilly‐Spong, Park, and Gross 2013), restless leg syndrome, and various complications like hepatic encephalopathy (Akahoshi et al. 2014), anxiety, stress, and depression (Demir and Saritas 2022; Mendes et al. 2014) are the factors affecting the quality of sleep. Several studies on different populations such as cancer patients have pointed out that intolerance of uncertainty has a negative effect on well‐being and causes sleep problems, anxiety, and depression (Lauriola et al. 2019; Wu et al. 2021; Panjwani, Millar, and Revenson 2021). This evidence offered an impetus for the present study and provided a rationale to study intolerance of uncertainty and the quality of sleep in LT recipients. To the best of our knowledge, there have not been any studies on intolerance of uncertainty and sleep quality in the relevant literature. Yıldız reported that intolerance of uncertainty was a risk factor of anxiety following liver transplantation (Yıldız 2021). A meta‐analysis emphasized that intolerance of uncertainty should be examined for the treatment of individuals at risk of anxiety (Miller and McGuire 2023). It is important to take account of intolerance of uncertainty and sleep quality in LT recipients likely to experience prospective uncertainty and sleeplessness due to multiple factors and with high risk of anxiety and depression to offer high quality nursing care (Yang et al. 2020; Yıldız 2021). Increased intolerance of uncertainty and decreased sleep quality can have a negative impact on daily lives of LT recipients (Carleton 2016a; Lauriola et al. 2019). Building on this observation, this study was directed towards examining intolerance of uncertainty and sleep quality in LT recipients. To this aim, answers to the following questions were sought.

1. What is the level of intolerance of uncertainty in LT recipients?
2. What is the quality of sleep in LT recipients?
3. Is there a difference in the mean score of LT recipients on intolerance of uncertainty in terms of their descriptive characteristics?
4. Is there a difference in the mean score of LT recipients on sleep quality in terms of their descriptive characteristics?
5. Is there a relation between the level of intolerance of uncertainty and the quality of sleep in LT recipients?

The results of the present study will fill a gap in the relevant nursing literature and play a role in the improvement of nursing care by taking account of intolerance of uncertainty and the quality of sleep.

## 2 Methods

### 2.1 Design and Participants

This study had a descriptive, cross‐sectional design. The study sample included 117 LT recipients fulfilling the inclusion criteria of the study and followed in the outpatient clinic of a LT center in Turkey between May and September in 2023. The inclusion criteria were being aged 18 years or older, accepting to participate in the study, minimum 1‐month and maximum 3‐years‐time elapsing after transplantation and the lack of a condition hindering communication. LT recipients with the prior diagnosis of psychological and mental problems (e.g., anxiety, depression, psychosis, etc.) were not included in the study.

The sample size was calculated by using G*Power 3.1.9.7 (Faul et al. 2007). The moderate effect size was utilized for multiple linear regression analysis (Cohen 1988). The sample size was found to be 92 based on the effect size of 0.15 (Cohen *f* ^2^ = 0.15), the margin of error of 5% and the power of 80% for the multiple linear regression analysis with five independent variables. Taking the possible loss of data (25%) into consideration, 117 LT recipients satisfying the inclusion criteria were included in the study sample (Bingöl et al. 2020).

### 2.2 Measurements

Data were collected with a sociodemographic and clinical features form, the Intolerance of Uncertainty Scale‐12 (IUS‐12) and the Pittsburgh Sleep Quality Index (PSQI).

### 2.3 Sociodemographic and Clinical Features Form

A sociodemographic and clinical features form was prepared by the researchers in light of the literature and it is composed of 20 questions about sociodemographic features (age, weight, height, gender, marital status, education, people staying with the LT recipients, employment status, income, etc.), transplantation‐related features (type of donor, time to transplantation, time elapsing after transplantation, posttransplant complications like pain, hernia, infection, bleeding, rejection, itching, and number of rehospitalizations after transplantation), and chronic diseases (Lim et al. 2023; Yıldız 2021; Zhu et al. 2020).

### 2.4 The IUS‐12

The IUS‐12 was developed by Carleton et al. in 2007 to determine the level of intolerance of uncertainty. It is a self‐report, five‐point Likert scale for individuals aged 16 years or older (1 = not true for me at all, 2 = very slightly true for me, 3 = somewhat true for me, 4 = very true for me, and 5 = completely true for me). The scale has two subscales, that is, prospective anxiety and inhibitory anxiety, and 12 items. The first item is reverse‐coded. The lowest and highest scores on the scale are 12 and 60, respectively. As the score on the scale increases so does the level of intolerance of uncertainty. Cronbach's *α* and test–retest reliability coefficient for the original IUS‐12 were reported to be 0.92 and 0.74, respectively (Carleton, Norton, and Asmundson 2007).

The validity and reliability of the IUS‐12 for the Turkish adults over 16 were tested by Sarıçam et al. in 2014 (Sarıçam et al. 2014). They tested the validity of the Turkish version of the scale through linguistic validity, content validity, and factor analysis. The confirmatory factor analysis performed to test the construct validity of the Turkish version revealed that the 12 items of the scale were loaded on two subscales (prospective anxiety and inhibitory anxiety) just like the original scale. The factor loads of the scale ranged from 0.55 to 0.87. The test–retest reliability of the scale was 0.74 and item‐total correlations ranged from 0.42 to 0.68. Cronbach's *α* was 0.88 for the overall scale, 0.84 for the subscale of prospective anxiety and 0.77 for the subscale of inhibitory anxiety (Sarıçam et al. 2014). Cronbach's *α* for the subscales of prospective anxiety and inhibitory anxiety in the Turkish version of the scale were reported to be 0.74 and 0.76, respectively, in LT recipients (Yıldız 2021) and Cronbach's *α* for the Turkish version of the scale in kidney transplant recipients was reported to be 0.91 (Menekli and Şentürk 2023). In the present study, Cronbach's *α* was 0.88 for the overall scale, 0.89 for the subscale of prospective anxiety and 0.97 for the subscale of inhibitory anxiety.

### 2.5 The PSQI

The PSQI was developed by Buysse et al. in 1989 to evaluate the sleep quality and sleep disorders experienced in the previous month (Buysse et al. 1989). The PSQI is a self‐assessment questionnaire used in the general population and in populations with different clinical diagnoses (Biyyala et al. 2023; Zhu et al. 2020; Lim et al. 2023). The index is a widely used and practical tool for examining sleep disorders in LT patients (Biyyala et al. 2023; Akahoshi et al. 2014; Bhat et al. 2015; Lim et al. 2023; Mendes et al. 2014; Reilly‐Spong, Park, and Gross 2013; Zhu et al. 2020). It has seven subscales: subjective sleep quality, sleep latency, sleep duration, habitual sleep efficiency, sleep disturbances, use of sleeping medication, and daytime disfunction. Some of the subscales have one item while others have a group of items. The PSQI is composed of 24 items, of which 19 are self‐report questions and the remaining five are answered by spouses or other relatives. The questions answered by spouses or relatives are not assigned points. The total score for each subscale can be 0–3. Adding the scores for all seven subscales gives the total score on the PSQI and ranges from 0 to 21. Scores of ≤ 5 indicate good quality sleep and scores of > 5 indicate poor quality sleep. The internal consistency of the original scale was 0.73, the test–retest reliability coefficient was 0.85 and Cronbach's *α* was 0.80. The sensitivity and specificity of the scale in distinguishing individuals with high quality sleep from those with poor sleep were 89.6% and 86.5%, respectively (Buysse et al. 1989).

The validity and reliability of the PSQI for the Turkish population were tested by Ağargün et al. in 1996. Many studies using the Turkish version of the PSQI have been conducted on organ transplant patients (Gençdal et al. 2020; Demir and Saritaş 2022; Yavlal, Aras, and Ulaş 2022). The validity of the index was achieved through linguistic validity, content validity and factor analysis. Cronbach *α* on the index was 0.70 and it was considered as a valid and reliable scale (Ağargün, Kara, and Anlar 1996). In the present study, Cronbach's *α* on the PSQI was 0.74.

### 2.6 Data Collection

Data were collected from LT recipients at an organ transplant center in Izmir, in the west part of Turkey. After the aim of the study was explained, oral and written informed consent of the participants was obtained. Data were collected using self‐report questionnaires. Data collection lasted for 20 min on average. After the LT recipients completed the survey, the researcher checked that all questions were answered and asked the participants to fill in the missing questions to reduce the amount of missing data. The organ transplant center provides services from 8 a.m. to 6 p.m. for 5 days a week. A nurse and four doctors work in the center. The doctors make preoperative preparations of LT recipients and donors and manage treatments of the recipients after transplantation. The nurse conducts the first interviews with the recipients and donors in the outpatient clinic, offers preoperative education to patients, monitors the levels of immunosuppressive medications and follow their side‐effects during follow‐up visits of the recipients and donors in the outpatient clinic and provides education when needed. The nurse also gives discharge education to the patients.

### 2.7 Ethical Considerations

Written permission was received from the hospital where this study was conducted (Date: 18.05.2023; Approval number: 2023/564). Ethical approval was obtained from the ethical board of a university (Date: 9.05.2023; Approval number: B.30.2.İEÜSB.0.05.05–20‐235). Permission was also requested from the researchers who developed the IUS‐12 and the PSQI. The LT recipients were informed about the aim of the study and their oral and written consent was obtained.

### 2.8 Data Analysis

Data were analyzed with Statistical Package for the Social Sciences 23.0 (IBM Inc., Armonk, NY, USA). The descriptive statistics utilized were number, percentage, mean, and standard deviation. Kolmogorov–Smirnov and Shapiro–Wilk tests were utilized to test the normality of the data. The parametric tests of independent groups *t* test and one‐way analysis of variance (ANOVA) were employed for normally distributed data and the non‐parametric tests of Mann–Whitney *U* test and Kruskal Wallis test were utilized for the data without a normal distribution (Polit and Beck 2017). Levene test was applied to test the assumption of homogeneity of variance for ANOVA. Normality assumption for regression analyses was assessed with Q–Q distribution plot (DeCarlo 1997). Effects of the significant variables on intolerance of uncertainty and sleep quality were examined with simple and multiple linear regression analysis. In the stage before performing linear regression, the distribution of the dependent variable was checked, which showed a normal distribution without outliers The relation between intolerance of uncertainty and sleep quality was examined with Pearson correlation analysis. The statistical significance was set at *p* < 0.05 (Polit and Beck 2017).

## 3 Results

### 3.1 Sociodemographic and Clinical Features of the Liver Transplant Recipients

The LT recipients were aged 22–78 years with a mean of 56.07 ± 11.15 years. Of all the recipients, 59% were male, 85.5% were married, and 76.1% were younger than 65 years. The mean age at the time of transplantation was 54.36 ± 11.13 years (min–max: 20–71 years). The mean time the recipients waited for transplantation was 59.17 ± 93.31 days (min–max: 7–540 days). The mean time elapsing after transplantation was 20.08 ± 23.73 months (min–max: 1–240). The mean body mass index of the recipients was 25.32 ± 3.48 kg/m^2^ (min–max: 18.36–36.26). Out of all the recipients, 58.10% were primary school graduates, 86.30% were unemployed, 90.6% had children, 40.20% were staying with their spouses, 64.10% were living in a small town, and 66.67% had an income lower than their expenses. Regarding their clinical features, 79.50% had one more chronic disease, 76.90% had a live donor, 71.80% had a donor from their relatives, and 42.73% had a donor from their first‐degree relatives. Besides, 53.00% were taking 6–10 medications and 91.45% experienced posttransplant complications. Of the recipients with posttransplant complications, 62.40% had nocturia. Also, 50.40% were not hospitalized after transplantation (Table 1).

**TABLE 1:** Sociodemographic and clinical features of the recipients (*n* = 117).

Variable	$\bar{X}\pm \mathrm{SD}$ (min–max)
Age (years)	56.07 ± 11.15 (22–78)
Age at transplant	54.36 ± 11.13 (20–71)
Waiting time for transplant (day)	59.17 ± 93.31 (7–540)
Time elapsing after transplant (month)	20.08 ± 23.73 (1–240)
Body mass index^a^	25.32 ± 3.48 (18.36–36.26)
Age groups	*n* (%)
< 65	89 (76.10)
65 ≤	28 (23.90)
Gender	
Female	48 (41.00)
Male	69 (59.00)
Marital status	
Single/widowed/divorced	17 (14.50)
Married	100 (85.50)
Educational level	
Primary education	68 (58.10)
High school	33 (28.20)
University	16 (13.70)
Working status	
Employed	16 (13.70)
Unemployed	101 (86.30)
Having children	
No	11 (9.40)
Yes	106 (90.60)
People staying with the recipients	
Alone	12 (10.30)
Spouse	47 (40.20)
Parents (father and/or mother)	12 (10.20)
Spouse and child	46 (39.30)
Place of residence	
City	28 (23.90)
Town	75 (64.10)
Village	14 (12.00)
Income	
Higher than expenses	7 (5.98)
Moderate	32 (27.35)
Lower than expenses	78 (66.67)
Chronic or accompanying diseases	
No	24 (20.50)
Yes	93 (79.50)
Donor type	
Cadaver	27 (23.10)
Living donor	90 (76.90)
Relationship with the donor	
Not a relative	33 (28.20)
Relative	84 (71.80)
Degree of the relationship with the donor	
Not a relative	33 (28.20)
1st degree (mother, father, child)	50 (42.73)
2nd degree (sibling, grandfather, grandmother, grandmother)	8 (6.83)
3rd degree (uncle, aunt, uncle, nephew)	12 (10.30)
4th degree (cousin, nephew's child)	4 (3.41)
Spouse and spouse's relative	10 (8.53)
Total number of medications used	
2–5	31 (26.50)
6–10	62 (52.99)
11–15	24 (20.51)
Complications after transplant	
No	10 (8.55)
Yes	107 (91.45)
Types of complications^b^	
Pain	40 (34.20)
Infection	21 (17.90)
Rejection	1 (0.90)
Itching	37 (31.60)
Hernia	21 (17.90)
Bleeding	1 (0.90)
Nocturia	73 (62.40)
Constipation	24 (20.50)
Diarrhea	20 (17.10)
Tremors	13 (11.11)
Numbness or weakness	12 (10.25)
Number of hospital readmissions after transplantation	
0	59 (50.40)
1	31 (26.50)
2	20 (17.10)
3 or more	7 (6.00)

^a^ Body mass index = body weight (kg)/height (m^2^).

^b^ There was more than one complication.

No statistically significant relationship was found between the mean score on the IUS‐12 (*r*_*p*_: 0.108) and the mean score on the subscale of inhibitory anxiety (*r*_*p*_: 0.002) and the mean score on the PSQI (*p* > 0.05). A statistically significant relationship was found between the mean score on the subscale of prospective anxiety and the mean score on the PSQI (*r*_*p*_: 0.193; *p* = 0.037).

### 3.2 Intolerance of Uncertainty and Sleep Quality in Liver Transplant Recipients

The mean score of the recipients on the IUS‐12 was 41.04 ± 13.11 (min–max: 12–60) (Table 2).

**TABLE 2:** The mean score on the Intolerance of Uncertainty Scale and the Pittsburgh Sleep Quality Index and sleep quality related factors of the recipients (*n* = 117).

Item	$\bar{X}\pm \mathrm{SD}$ (min–max)
Mean IUS‐12 total score	41.04 ± 13.11 (12–60)
Prospective anxiety	25.81 ± 7.24 (7–35)
Inhibitory anxiety	15.23 ± 6.81 (5–25)
Mean PSQI score	7.33 ± 3.57 (0–21)
	*n* (%)
PSQI ≤ 5 (good sleepers)	41 (35.00)
PSQI > 5 (poor sleepers)	76 (65.00)
Time to fall asleep (minutes)	31.33 ± 23.51 (5‐120)
Time spent in bed (hours)	7.30 ± 1.11 (5‐11)
Sleep duration (hours)	5.83 ± 1.01 (3‐9)
	*n* (%)
Sleep duration (hours)	
≤ 5	44 (37.60)
5 <	73 (62.40)
Sleeping difficulties experienced at least three nights per week	
Waking up in the middle of the night or early morning	62 (53.00)
Getting up to use the bathroom	67 (57.30)
Experiencing pain	4 (3.40)
Coughing or loud snoring	2 (1.70)
Nightmares	3 (2.60)
Feeling too hot	2 (1.70)
Feeling too cold	5 (4.30)
Being unable to breathe comfortably	3 (2.60)
Self‐rating of overall sleep quality	
Very good	14 (11.96)
Fairly good	58 (49.58)
Fairly bad	33 (28.20)
Very bad	12 (10.26)
Sleep efficiency (%)	
≥ 85.0	48 (41.02)
75–84	37 (31.62)
65–74	22 (18.81)
< 65	10 (8.55)
Use of sleep medications	
None	102 (87.17)
< 1 per week	6 (5.13)
1 or 2 per week	9 (7.70)
Sleep latency (minutes)	
0–15	14 (12.00)
16–30	30 (25.60)
31–60	41 (35.00)
> 60	32 (27.40)

Abbreviations: IUS‐12= Intolerance of Uncertainty Scale‐12; PSQI= Pittsburgh Sleep Quality Index; Sleep efficiency (%) = (number of hours slept/numbers of hours spent in bed) × 100; $\bar{X}\pm \mathrm{SD}$ = Mean ± standard deviation.

The mean score of the recipients on the PSQI was 7.33 ± 3.57 (min–max: 1–21). Also, 65% of the recipients obtained a score of over 5 on the PSQI and it took 5–120 min for the recipients to fall asleep with a mean of 31.33 ± 23.51 min. The mean time spent in bed was 7.30 **±** 1.11 h (min–max: 5–11 h) and the total sleep duration was 5.83 **±** 1.01 h (min‐max: 3–9 h). Besides, 62.4% of the recipients slept for more than 5 hours and 62.4% spent more than half an hour to fall asleep. A total of 58.98% of the recipients had sleep efficiency of less than 85% (the ratio of the actual sleep period to time spent in bed) and 87.17% did not take any sleeping pills in the previous month. Concerning the reasons for waking up at midnight or early in the morning in the previous month, 53% experienced sleeping problems/insomnia, 57.3% used the bathroom, 3.4% experienced pain, 1.7% coughed or snored loudly, 2.6% had a nightmare, 1.7% had the feeling of excessive heat, 4.3% had the feeling of excessive cold, and 2.6% could not breathe comfortably. A total of 38.46% of the recipients reported that their sleep quality was fairly poor or very poor (Table 2).

### 3.3 Factors Affecting the Recipients' Intolerance of Uncertainty and Sleep Quality

**FIGURE 1:**
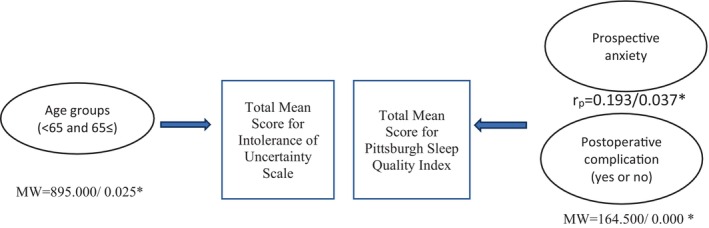
Differences in the mean scores on the Intolerance of Uncertainty Scale and the Pittsburgh Sleep Quality Index in terms of sosyo‐demographic and clinic characteristics *p < 0.05. MW = Mann Whitney *U* test, *r*_*p*_ = Pearson correlation analysis.

The mean scores on the IUS‐12 significantly differed in terms of age groups (< 65 years versus 65 ≤ years) (*MW*: 895.000; *p* = 0.025) (Figure 1). However, the mean score on the IUS‐12 did not differ significantly with respect to gender, marital status, education, employment status, having children, people staying with the recipients, place of living, income, having chronic diseases, type of donor, the degree of the relation with the donor, the total number of medications used, experiencing posttransplant complications, and rehospitalization after transplantation (*p* > 0.05).

The predictive value of age, which was found to be statistically different in the descriptive analyses (Mann–Whitney *U* test), on intolerance of uncertainty was examined using simple linear regression analysis. The regression model created was statistically significant (*F* (1.115) = 6.217, *p* = 0.014). Age was found to be a significant predictor of intolerance of uncertainty (*p* < 0.05). Age explained 5.1% of the variance in intolerance of uncertainty (*R* ^*2*^ = 0.051) (Table 3). One unit of an increase in age (1 year) caused a decrease in the level of intolerance of uncertainty by 0.266 unit (Table 3).

**TABLE 3:** The effect of age on intolerance of uncertainty.

Independent variables	Unstandardized coefficients		Standardized coefficients	*t*	*p*	95% CI	
	*B*	SE	*Β*			Lower bound	Upper bound
(Constant)	55.973	6.104		9.170	0.000	43.882	68.064
Age	−0.266	0.107	−0.226	−2.493	0.014	−0.478	−0.055

*Note:* Dependent variable: Intolerance of Uncertainty Scale Score. Durbin–Watson = 1.990; *F* = 6.217; *p* < 0,005; *R* = 0.226; *R* ^2^ = 0.051; Adjusted *R* ^2^ = 0.043.

Abbreviations: *β* = standardized regression coefficient; *B* = unstandardized coefficients; CI = confidence interval; SE = standard error.

* *p* < 0.05.

The mean scores on the PSQI significantly differed in terms of experiencing complications (*MW*: 164.500; *p* = 0.000) (Figure 1). However, the mean score on the PSQI did not significantly differ with regard to age, gender, marital status, education, employment status, having children, individuals staying with the recipients, place of living, income, having chronic diseases, type of donor, relation with the donor, number of medications used, and rehospitalization after transplantation (*p* > 0.05).

Multiple linear regression analysis was used to examine whether post‐transplant complication status, which was found to be statistically different in the descriptive analyses, and prospective anxiety, which is correlated with each other, predicted sleep quality. The regression model created was statistically significant (*F* (2.114) = 9.483, *p* = 0.000). Experiencing complications after transplantation explained 14.3% of the variance in the PSQI score (*R* ^2^ = 0.143) and was found to be significantly predictive of sleep quality (*p* < 0.001). Sleep quality was 4170 points worse in the recipients experiencing postoperative complications. Prospective anxiety was not predictive of sleep quality (Table 4).

**TABLE 4:** The effect of experiencing postoperative complications on the sleep quality.

Independent variables	Unstandardized coefficients		Standardized coefficient	*t*	*p*	95% CI	
	*B*	SE	*Β*			Lower bound	Upper bound
(Constant)	1.573	1.448		1.087	0.279	−1.294	4.441
Prospective anxiety	0.075	0.043	0.153	1.746	0.083	−0.010	0.161
Experiencing complications after transplant (*R* = no)	4.170	1.114	0.327	3.744	0.000	1.963	6.377

*Note:* Dependent variable: Pittsburgh Sleep Quality Index Score. Durbin–Watson = 1.699; *F* = 9.483; *p* < 0.001; *R* = 0.378; *R* ^2^ = 0.143; Adjusted *R* ^2^ = 0.128.

Abbreviations: *β* = standardized regression coefficient; *B* = unstandardized coefficients; CI = confidence interval; SE = standard error.

* *p* < 0.01.

## 4 Discussion

Researching the quality of sleep and intolerance of uncertainty and the relation between them in LT recipients can contribute to the improvement of physical and psychological well‐being and increase the quality of healthcare services. In the present study, the quality of sleep did not have a relation with intolerance of uncertainty and inhibitory anxiety, but was related to prospective anxiety in the LT recipients. It is striking that inhibitory anxiety and intolerance of uncertainty had no relation with the quality of sleep. Inhibitory anxiety means that the anxiety experienced by individuals during uncertainties increases to the extent that it disrupts their daily lives or functioning (Demirdaş and Bozdoğan 2013). The lack of a relationship between inhibitory anxiety and the quality of sleep in the present study can be attributed to activation of inhibitory anxiety in certain situations of daily life and its possible decrease during more passive situations like sleep. It can be useful to examine the long‐term effects of posttransplant inhibitory anxiety on LT recipients (e.g., social status and academic performance). Since there have not been any studies on the relation between the quality of sleep and intolerance of uncertainty in LT recipients, further studies could focus on that relation.

In the present study, the IUS‐12, utilized to determine the level of intolerance of uncertainty in the LT recipients, does not have a cut‐off point. Still, it can be suggested that the recipients had high levels of intolerance of uncertainty (41.04 ± 13.11; min–max: 12–60). To our knowledge, there has been only one study that examined the levels of intolerance of uncertainty in LT recipients (Yıldız 2021). The study revealed a moderate level of intolerance of uncertainty in the recipients (34.42 ± 8.35) (Yıldız 2021). The reason why the LT recipients in the present study had a higher level of intolerance of uncertainty when compared to those in Yılmaz's study can be increasing economic challenges in Turkey year by year. In addition, having an income lower than expenses, which was true for most of the recipients, gradual privatization and commercialization of the healthcare system, high costs of organ transplantations and the necessity for the recipients to allocate an additional budget for follow‐up appointments might have affected intolerance of uncertainty. Several studies have shown that the quality of life of LT recipients living in countries with socio‐economic difficulties considerably decreases and that they are more likely to experience anxiety and depression (De Simone et al. 2024; Sgrò et al. 2023). Moreover, a lack of structured patient follow‐up systems in Turkey (Köse 2023) and limited education before and after transplantation might have affected intolerance of uncertainty. It was revealed in the literature that LT recipients had non‐adherence to treatment and a low quality of life in cases of insufficient information (Moayed et al. 2019; Taher et al. 2021) since fear of the unknown was the core factor underlying intolerance of uncertainty (Carleton 2016b). In light of the findings of the present study, nurses offering care to LT recipients could be recommended to focus on uncertainties in the recipients' mind. Besides, it can be suggested that nurses should identify and fulfill information needs of the recipients during the caregiving process and prioritize their emotions and opinions. Moreover, researchers and transplantation teams should evaluate the factors likely to affect intolerance of uncertainty by adopting a multidisciplinary approach.

Since globally individuals aged 65 years and older are considered as elderly (OECD 2024), in the present study, the mean scores on intolerance of uncertainty were compared between the individuals younger than 65 years and those aged 65 years and older. The former group was shown to experience high intolerance of uncertainty. Age was found to affect uncertainty and as age decreased, intolerance of uncertainty increased. Indeed, age was responsible for 5.1% of the variance in the mean IUS‐12 score. There have not been any studies in the literature to examine the effect of age on the level of intolerance of uncertainty in organ transplant recipients. Although elderly patients having liver transplantation have a higher risk of comorbidities (Durand et al. 2019), the younger recipients were found to have higher intolerance of uncertainty in the present study. It may be that young recipients face many difficulties compared to their peers, cannot attend as many social activities as their peers, experience difficulty in their relationships with their friends, relatives and colleagues and feel more worries about their future plans. On the other hand, recipients over 65 years old may associate their transplant‐related health problems with aging and consider them as normal. Based on the results of the study, it can be recommended that further studies should focus on psychosocial needs of young people and young adults receiving LT.

In the current study, 65% of the LT recipients had a poor sleep quality and a mean PSQI score of over the cut‐off point 5 (7.33 ± 3.57). Congruent with this finding, LT recipients from Turkey (PSQI score: 6.10 ± 1.41) (Demir and Saritaş 2022), China (PSQI score: 6.57 ± 4.28) (Zhu et al. 2020), and Korea (PSQI score: 7.16 ± 4.15) (Lim et al. 2023) were reported to have a poor sleep quality. Studies in Brazil and Japan also revealed a poor sleep quality following liver transplantation (Akahoshi et al. 2014; Bhat et al. 2015; Mendes et al. 2014; Reilly‐Spong, Park, and Gross 2013). The prevalence of the poor sleep quality has been reported to range from 40.3% to 72% (Akahoshi et al. 2014; Bhat et al. 2015; Lim et al. 2023; Mendes et al. 2014; Reilly‐Spong, Park, and Gross 2013; Zhu et al. 2020). Clearly, poor sleep is a commonly experienced symptom after liver transplantation (Biyyala et al. 2023). Since only the sleep quality was examined following transplantation, further studies with control groups are needed to examine the factors that can influence sleep patterns of the patients before and after transplantation. The results of the study are important for health professionals providing care after liver transplantation since they shed light on the healing process of sleep disorders and sleep quality after transplantation.

Consistent with the literature (Lim et al. 2023; Zhu et al. 2020), only 12.8% of the LT recipients in the current study were found to take sleeping pills despite their poor sleep quality. This may be because they are aware of the fact that sleeping pills interact with immunosuppressive medications given after liver transplantation (Reilly‐Spong, Park, and Gross 2013). Moreover, sleeping pills cannot be obtained without prescription, which might have reduced their use.

In the present study, 62.4% of the LT recipients reported that they could not fall asleep for 30 min after they lay in their bed. Unlike the present study, prior studies have shown that the rate of the recipients unable to sleep in 30 min varied from 24.8% to 38.7% (Lim et al. 2023; Reilly‐Spong, Park, and Gross 2013; Zhu et al. 2020). The mean duration of sleep was found to be 5.83 **±** 1.01 h (min–max: 3–9 h) in the present study. It is reported in the literature that the total sleep duration in LT recipients ranged from 6.1 to 7 h (Bhat et al. 2015; Lim et al. 2023; Mendes et al. 2014). Delayed onset of sleep and short duration of total sleep can be attributed to two factors. First, even if prospective anxiety did not predict the quality of sleep, there is a relation between prospective anxiety and the quality of sleep. It can be considered that individuals with prospective anxiety have a constantly active mind, which may delay the onset of sleep or cause disruptions of sleep. Second, posttransplant complications may play a role. In the current study, the recipients experiencing complications like nocturia, pain, and itching had a poorer sleep quality than those without posttransplant complications. Indeed, experiencing posttransplant complications explained 14.3% of the mean PSQI score. Reilly‐Spong et al. reported that experiencing pain was a factor strongly related to a poor quality of sleep (Reilly‐Spong, Park, and Gross 2013). Yıldız showed that 35.6% of the LT recipients had various posttransplant complications like infections and bleeding (Yıldız 2021). The most frequent complications detected in the present study were nocturia (62.40%) and pain (34.20%). Furthermore, more than half of the recipients were found to wake up at midnight or early in the morning. This finding suggests that nocturia and pain are important indicators of poor sleep in LT recipients. In line with the finding of the present study, several studies have revealed that nocturia is one of the side‐effects of immunosuppressive therapy after organ transplantation (Bhat et al. 2015; Demir and Saritaş 2022; Lim et al. 2023; Mendes et al. 2014; Zhu et al. 2020). Nurses and other health professionals should evaluate posttransplant complications experienced by transplant recipients thoroughly, determine their causes and provide solutions to them.

## 5 Limitations

Since this study was performed with LT recipients in one organ transplant center, its results cannot be generalized to all LT recipients. In addition, the study examined the post‐transplant period by adopting a cross‐sectional design. Therefore, the change in intolerance to uncertainty and sleep quality over time before and after liver transplantation could not be determined. Therefore, it may be recommended that multicenter, long‐term studies with a control group be conducted in the future. Factors related to psychological status and lifestyle can affect health behaviors and outcomes of individuals. However, since the current study specifically focused on the variables found to have a relation in descriptive analyses (age, experiencing complications, and prospective anxiety), these types of variables could not be examined.

## 6 Conclusions and Implications for Practice

To conclude, the LT recipients had high intolerance of uncertainty, age was predictive of uncertainty, and younger LT recipients had higher intolerance of uncertainty. Moreover, the LT recipients had a poor sleep quality and experiencing posttransplant complications worsened the quality of sleep. There was no relation between the quality of sleep and intolerance of uncertainty and inhibitory anxiety. Although there was a relationship between sleep quality and prospective anxiety, it was not a significant predictor of sleep quality.

It can be recommended that health professionals should evaluate LT recipients thoroughly, frequently give them inform and support them after LT to reduce intolerance of uncertainty and improve the quality of sleep. Furthermore, they could develop technology‐assisted education programs that can be easily accessed and help LT recipients to overcome immunosuppressive therapy‐related complications like nocturia, pain, and itching after transplantation. While comorbidities should be closely followed in LT recipients aged over 65 years, young LT recipients should not be neglected. It can be suggested that the relation between the level of intolerance of uncertainty and sleep quality should be examined in different organ transplant populations.

## Author Contributions

**Özgül Karayurt:** supervision, writing – review and editing, visualization, writing – original draft, conceptualization, investigation. **Murat Kiliç:** supervision, writing – review and editing, conceptualization. **Gülay Aksu Kul:** conceptualization, data curation. **Adile Savsar:** conceptualization, investigation, writing – original draft, methodology, visualization, writing – review and editing, data curation.

## Ethics Statement

Written permission was received from the hospital where this study was conducted (Date: 18.05.2023; Approval number: 2023/564). Ethical approval was obtained from the Health Sciences Research Ethics Committee of Izmir University of Economics (Issue: 2023/B.30.2.IEUSB.0.05.05–20‐235).

## Conflicts of Interest

The authors declare no conflicts of interest.

## Data Availability

The data that support the findings of this study are available on request from the corresponding author. The data are not publicly available due to privacy or ethical restrictions.
